# A multimodal image guiding system for Navigated Ultrasound Bronchoscopy (EBUS): A human feasibility study

**DOI:** 10.1371/journal.pone.0171841

**Published:** 2017-02-09

**Authors:** Hanne Sorger, Erlend Fagertun Hofstad, Tore Amundsen, Thomas Langø, Janne Beate Lervik Bakeng, Håkon Olav Leira

**Affiliations:** 1 Department of Circulation and Imaging, Faculty of Medicine, Norwegian University of Science and Technology (NTNU), Trondheim, Norway; 2 Department of Thoracic Medicine, St Olavs Hospital, Trondheim University Hospital, Trondheim, Norway; 3 Department of Medicine, Levanger Hospital, North-Trøndelag Health Trust, Norway; 4 Department of Medical Technology, SINTEF Technology and Society, Trondheim, Norway; 5 Norwegian National Advisory Unit for Ultrasound and image-guided therapy, St. Olavs Hospital, Trondheim, Norway; Postgraduate Institute of Medical Education and Research, INDIA

## Abstract

**Background:**

Endobronchial ultrasound transbronchial needle aspiration (EBUS-TBNA) is the endoscopic method of choice for confirming lung cancer metastasis to mediastinal lymph nodes. Precision is crucial for correct staging and clinical decision-making. Navigation and multimodal imaging can potentially improve EBUS-TBNA efficiency.

**Aims:**

To demonstrate the feasibility of a multimodal image guiding system using electromagnetic navigation for ultrasound bronchoschopy in humans.

**Methods:**

Four patients referred for lung cancer diagnosis and staging with EBUS-TBNA were enrolled in the study. Target lymph nodes were predefined from the preoperative computed tomography (CT) images. A prototype convex probe ultrasound bronchoscope with an attached sensor for position tracking was used for EBUS-TBNA. Electromagnetic tracking of the ultrasound bronchoscope and ultrasound images allowed fusion of preoperative CT and intraoperative ultrasound in the navigation software. Navigated EBUS-TBNA was used to guide target lymph node localization and sampling. Navigation system accuracy was calculated, measured by the deviation between lymph node position in ultrasound and CT in three planes. Procedure time, diagnostic yield and adverse events were recorded.

**Results:**

Preoperative CT and real-time ultrasound images were successfully fused and displayed in the navigation software during the procedures. Overall navigation accuracy (11 measurements) was 10.0 ± 3.8 mm, maximum 17.6 mm, minimum 4.5 mm. An adequate sample was obtained in 6/6 (100%) of targeted lymph nodes. No adverse events were registered.

**Conclusions:**

Electromagnetic navigated EBUS-TBNA was feasible, safe and easy in this human pilot study. The clinical usefulness was clearly demonstrated. Fusion of real-time ultrasound, preoperative CT and electromagnetic navigational bronchoscopy provided a controlled guiding to level of target, intraoperative overview and procedure documentation.

## Introduction

Lung cancer is the most frequent cancer type in the world. The overall prognosis is poor [1]. Early and correct identification of limited disease with potential to cure is crucial for long-term patient survival. Thorough investigation of the mediastinum is fundamental, since surgery is ruled out in case of locoregional lymph node metastasis [1].

Recent lung cancer guidelines suggest endobronchial ultrasound transbronchial needle aspiration (EBUS–TBNA) as the method of choice for detecting mediastinal lymph node metastasis [1]. EBUS-TBNA is a minimally invasive, gentle and available method that can be performed outpatient. EBUS-TBNA is therefore widely used to determine the stage of disease, select patients with curative potential and establish correct individual treatment strategies.

The EBUS bronchoscope used in lung cancer staging has a convex ultrasound (US) probe at the tip that scans a 60° image sector parallel to the bronchoscope shaft. The US signal can detect lymph nodes several centimeters outside the airway wall. Endoscopic view is used for airway orientation while advancing towards the region of interest. The US probe is then used for exact localization and visualization of the target, and real-time sampling guidance. A transbronchial needle system is inserted through the working channel of the bronchoscope, and used to aspirate a transbronchial specimen by intermittent suction and agitation of the needle. EBUS-TBNA has a good diagnostic rate [2–6]. Despite its advantages as a real-time image-guiding method, EBUS can still pose challenges to the bronchoscopist. The EBUS image quality is at times reduced due to artifacts and poor contact between the US probe and tracheobronchial wall. The US field of view is small, and provides no information on out of plane structures, so the operator has to rely on preoperative images (most often CT) to memorize the patient’s anatomy. Radiology images are not directly linked to the patient, and a period of time may pass between CT acquisition and endoscopy. Anatomical shifts due to tumor expansion, lung collapse or fluid accumulation occuring in the meantime can therefore be overlooked. Intraoperative movements due to breathing, heart beats and pressure from the bronchoscope itself also cause a real risk of sampling inaccuracy [7]. Since bronchoscopy images are rarely stored in medical records, sampling errors may pass unnoticed.

Another challenge is that mediastinal lymph nodes are organized in closely positioned lymph node stations [8], separated by anatomical landmarks that can be hard to identify without EBUS experience. Exact knowledge of which lymph node stations are involved in lung cancer is crucial, since this impacts clinical decision-making and patient survival [8]. New and better strategies to increase diagnostic confidence, yield and safety, reduce procedure time and improve operator learning curves are therefore called for.

Improvement of EBUS-TBNA has been explored by using advanced image-guiding technology, such as electromagnetic navigated bronchoscopy (ENB) [9, 10]. ENB has successfully supported access to mediastinal lymph nodes in previous studies [11, 12].

Combined EBUS and ENB has also been explored with commercial systems, but only using radial array US probes that are designed primarily for the peripheral airways. The improvement in diagnostic yield has not been enough to justify adoption of combined radial probe EBUS/ENB technology [13, 14]. The aim of this study was to demonstrate the feasibility of a multimodal image guiding system using electromagnetic navigation for ultrasound bronchoschopy in humans.

## Materials and methods

The study was performed with approval from the Regional Committees for Medical and Health Research Ethics (REC South East, project number 2010/3385), in compliance with ethical practices, according to the principles expressed in the Declaration of Helsinki, and recorded in the ClinicalTrials.gov Registry (“Automatic CT-to-patient Registration During Navigated Bronchoscopy and EBUS”, registration number NCT02493023 [15, 16]).

### Experimental equipment

#### Software

Our group has recently developed a research navigation system for convex probe EBUS-TBNA, successfully demonstrated in preclinical experiments [10]. The system consists of a prototype EBUS-bronchoscope and a multi-purpose image guidance software (CustusX, SINTEF, Norway) that has previously been implemented in a range of clinical areas [15, 17]. The software was recently modified to support EM navigation of bronchoscopy procedures and made freely available in open source code in 2015 (www.custusx.org). Shortly after the current study was initiated, a simplified navigation software tailor-made for bronchoscopy was released [18].

#### Electromagnetic tracking and bronchoscopes

**Fig 1:**
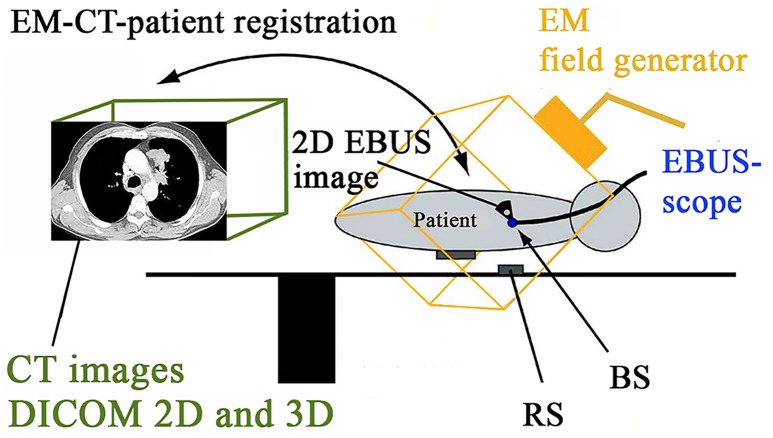
Schematic operating room setup during navigated EBUS-TBNA. Preoperative images in DICOM format were imported into the navigation software, and matched to the patient’s position during bronchoscopy (EM-CT-patient registration). When maneuvering the bronchoscope within the electromagnetic tracking (EM) field, the position of the bronchoscope sensor (BS) and EBUS images could be acquired in the navigation system. A reference electromagnetic sensor (RS) was attached on the table.

**Fig 2:**
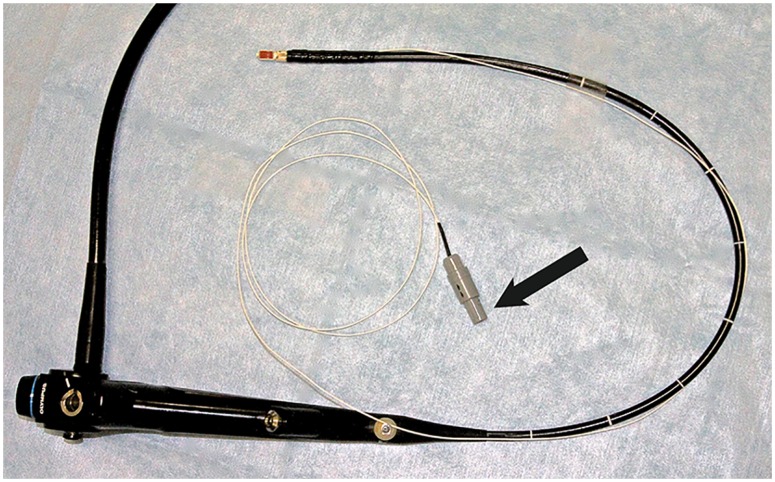
Experimental EBUS bronchoscope. The sensor for electromagnetic tracking was attached close to the convex probe in the tip of the bronchoscope. The connector to the sensor (arrow) was plugged into the control unit of the electromagnetic tracking system.

To track the position of bronchoscopy tools inside the body, we used the generic Aurora^®^ EM tracking system by NDI (Northern Digital Inc (NDI), Waterloo, ON, Canada)[19, 20]. A transmitter box set up an EM field around the patient’s chest during endoscopy (Fig 1). An EM tracking sensor (Aurora 6DOF Probe, Straight Tip, Standard) was attached close to the tip of a convex probe EBUS bronchoscope (Olympus BF UC160F, Olympus, Tokyo, Japan) (Fig 2). A regular video bronchoscope (Olympus BF Q160, Olympus, Tokyo, Japan), also equipped with a position tracking sensor, was used for visual inspection of the airways and navigation system calibration before the procedure [16].

#### OR setup

The OR setup included a conventional EBUS-rack with a video processing unit (Olympus CV-190, Olympus, Tokyo, Japan), light source (Olympus CLV-190,) and US processor (Olympus EUS Exera EU-C60). A cable from the US processor allowed EBUS image import to the navigation system [15]. The navigation set-up included a monitor, a computer running the navigation software, and an EM positioning system control unit. The EM field generator was mounted on the operating table close to the patient. The position sensor on the research bronchoscope was connected to the EM control unit. The exact position of the bronchoscope sensor (Fig 2) could then be displayed with fused real-time EBUS images and three-dimensional (3D) image maps made from the patient’s own preoperative CT. The monitor with navigation visualizations was placed in front of the operator. A schematic overview of the OR setup is presented in Fig 1.

#### Multimodal image fusion

**Fig 3:**
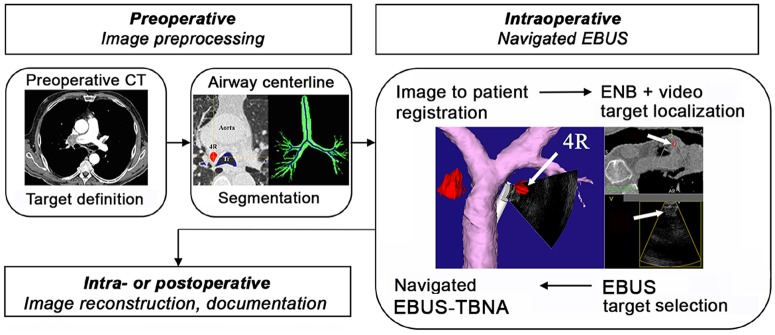
Electromagnetic navigated EBUS-TBNA, procedure workflow. Preoperative preparations included target definition, CT model extraction and image import into the navigation software. During the EBUS procedure, image-to-patient registration was performed using an automatic algorithm in the navigation software. A combination of video, electromagnetic navigated bronchoscopy (ENB) and EBUS was used for target localization and confirmation before EBUS guided fine needle sampling. A variety of options existed for image reconstruction during the procedure or postoperatively.

**Fig 4:**
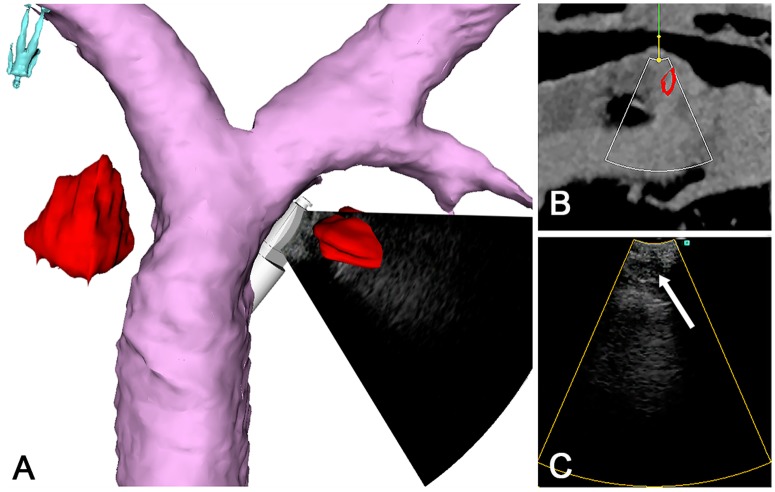
Graphical user interface, patient 4. (A) The CT models of the central airways, a 4R and a 4L lymph node target, the tip of the EBUS-scope and the real-time EBUS image. (B) The corresponding image plane from preoperative CT. The thin white sector represents the exact position of the EBUS image. (C) Regular, real-time EBUS image, showing the 4R lymph node (arrow).

Correct position and orientation match between the patient, the patient’s preoperative CT images, the bronchoscope and the US images was provided by a series of steps. While developing the bronchoscope prototype, a calibration of the US probe was performed, determining the constant relationship between the sensor and the US image [21]. Preoperative CT images were imported into the navigation system and automatically aligned to the patient during the initial phase of bronchoscopy (Figs 1 and 3) [15, 16]. The *image-to-patient registration* process is described in more detail in the intraoperative experiments section. Live images from the EBUS probe were exported from the US processor to the navigation system by an analogue-to-digital converter. It was then possible to display fused pre- and intraoperative images together with the position of the bronchoscope in the same navigation scene (Fig 4).

### Patient study

#### Patients

We performed a prospective, non-randomized enrollment of patients referred to the Department of Thoracic Medicine for lung cancer diagnosis and staging. Non-pregnant patients >18 years with suspected mediastinal lymph node metastasis were asked to participate in the study. Written, informed consent was obtained by the investigating physician at the first visit in the Department of Thoracic Medicine. All study procedures were performed under conscious sedation with midazolam and alfentanil according to local standards (Fig 3), by the main author (H. S.). Afterwards, all patients were monitored in hospital according to local guidelines, for at least two hours.

#### Preoperative image processing

**Fig 5:**
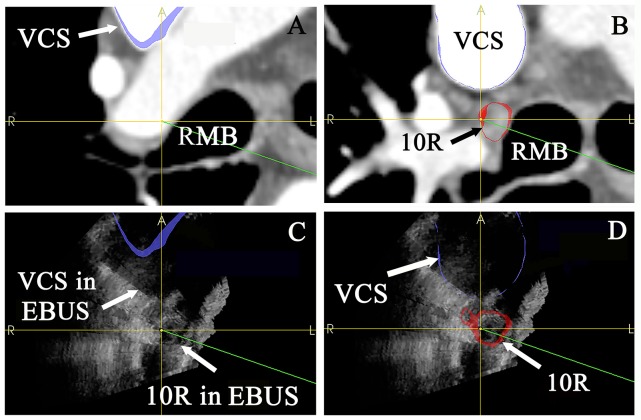
Navigation system accuracy, patient 3. (C, D) Orthogonal EBUS planes were projected on top of corresponding planes from the segmented CT models. Here, the axial plane is chosen for method illustration. (B, D) The EBUS and CT position of a 10R lymph node and the superior vena cava after manual shift correction. The resulting position deviation between EBUS and CT was combined for three planes, representing the navigation system accuracy. RMB = Right main bronchus. VCS = Superior vena cava.

Preoperative thoracic CT images were available for all patients, acquired at the referring hospitals. There were no predefined requirements to the CT protocol. CT slice thickness was between 1.0–3.5 mm. All CT’s were interpreted by a thoracic radiologist at the study hospital. The CT images were then imported into the navigation software in DICOM format. The target lymph nodes for TBNA were defined by the performing operator. CT models were created and used for intraoperative orientation and postoperative accuracy measurements (Figs 4 and 5). The airway models were automatically extracted from CT by the navigation system [22], while CT models of targets and selected vessels were segmented using ITK-SNAP software (www.itksnap.org), and then imported into CustusX [17, 20].

#### Intraoperative experiments

The experiment was conducted as part of a regular EBUS procedure in a standard bronchoscopy suite. Two different bronchoscopes were therefore used, first a video bronchoscope for a complete visual inspection of the airways, and then an EBUS bronchoscope for US imaging and TBNA guidance. Both bronchoscopes were continuously tracked in the EM field surrounding the patient.

The registration of preoperative CT images to the patient’s position on the OR table was performed during the initial phase of bronchoscopy. The experimental video-bronchoscope with an attached position sensor and a novel, automatic image-to-patient registration algorithm were used (Figs 1–4) [15, 16]. The navigation system acquired the pathway of the bronchoscope while it was maneuvered through the airways applying topical anesthetics. The navigation software then matched the bronchoscope pathway to the airway centerline automatically extracted from the patient’s CT data [22] (Fig 3). After inspection of the lobar and segmental bronchi the video bronchoscope was replaced by the experimental EBUS-bronchoscope.

The EBUS bronchoscope was advanced inside the trachea and main bronchi. The approximate level of the target lymph node was located using combined video and EM navigation guidance. The ultrasound images were displayed in the navigation system. Needle sampling was assisted by the fused US and CT images. EBUS was used to verify the correct lymph node level, to select the predefined lymph node from others in close vicinity, and to confirm target position during TBNA. Conventional cytology needles were used for sampling. All position measurements (targets, vessels, the position of the EBUS-probe and the sequence and localization of TBNA’s) were stored in the navigation system, together with EBUS recordings [15].

### Clinical outcomes

#### Adequacy of samples

The adequacy of samples was recorded according to on-site evaluation by a cytopathologist. A specimen was considered adequate if > 40 lymphocytes were identified per high power field of view in the light microscope. Overall adequacy of samples was reported per lymph node.

#### Procedure time

Procedure time was measured from first passage of the video-bronchoscope through the vocal cords, to the final withdrawal of the experimental EBUS-bronchoscope after all sampling was completed.

#### Adverse or unexpected events

Procedure related adverse events were recorded by the performing bronchoscopist before the patient was discharged, and grouped into the following categories: pneumothorax, excess bleeding, cardiovascular and respiratory adverse events and others.

### Technical outcomes

#### Image fusion and visualization

Overall navigation system functionality was defined as successful fusion and display of multiple image modalities in the OR: the patient’s CT data, segmented CT models, and real-time EBUS images. Image fusion and visualization were not subject to quality assessment, rather considered successful if possible at all in patients.

#### Bronchoscope guiding

Bronchoscope guiding was considered feasible if the navigation system was able to display the real-time position of the tracked EBUS probe throughout the procedure. A subjective statement was given by the operator on the system’s usefulness in localizing the region of interest, identifying bronchoscope position and sampling.

#### Navigation system accuracy

Navigation accuracy was calculated postoperatively [15]. EBUS recordings were used to define the true position of target lymph nodes at the time of image acquisition, and were compared to the image position of the same lymph node’s 3D CT model. The position coordinates of the targets in EBUS and CT volumes were retrieved from the navigation system. A manual alignment, or shift correction, was then performed to find the position deviation between CT and EBUS, representing navigation accuracy: Three orthogonal planes (x, y and z) of the EBUS volumes were used to overlay the corresponding planes in segmented CT models (Fig 5). The mean and median navigation accuracy was then calculated by combining the shift correction in each plane. All target positions in EBUS and CT were validated by two persons to reduce operator-dependent error, either by comparing to the position of a vessel (Fig 5), or by replaying the 2D EBUS recording sequence on top of the corresponding orthogonal US image plane.

### Statistical analysis

Simple mean and median were calculated for the navigation accuracy and the clinical outcomes.

## Results

Four patients aged 69–75 years were enrolled in the study September 2015. EM navigation and TBNA were successfully performed in six mediastinal lymph nodes (Table 1). Five of the lymph nodes were completely visualized and could be used for accuracy measurements (in a total of 11 EBUS recordings) (Table 2).

**Table 1:** Navigated EBUS: Patient baseline data and clinical outcomes.

Patient	Lymph node station	Lymph node size in CT (mm)	TBNA adequacy	Result ROSE	Procedure time (min:sec)	Adverse events
1	4R	30	4/4	SCLC	16:14	None
2	4L	16	2/2	SCLC	15:00	None
3	10R	11	2/4	Lymphatic tissue	25:05	None
4	4R	7	1/3	Lymphatic tissue	42:00	None
	7	10^1^	2/3			
	4L	10	2/3			
	n = 6	Mean 14.0	13/19		Mean 24:38	
		Median 10.5	6/6 (100%)^2^		Median 21:25	

^1^ Not possible to assess the complete lymph node with EBUS, excluded from accuracy calculations

^2^ Adequacy of samples per lymph node

ROSE = Rapid on-site cytological evaluation. SCLC = Small cell lung carcinoma.

**Table 2:** Navigated EBUS: Technical outcomes.

Patient	Lymph node station	Plane offset			Total navigation error (mm)	Structure used to validate target position
		X	Y	Z		
1	4R	0	-3.6	5.8	6.8	
2	4L	-9.8	1.4	5.6	11.4	AA
		-12.5	0.8	3.5	13.0	
		-10.3	0.8	5.4	11.7	
3	10R	-1.6	-8.7	8.5	12.3	PA and AV
		-3.7	-8.3	6.2	11.0	
		-1.2	-9.1	15	17.6	
4	4R	2.7	-2.5	7.3	8.2	2D US recording
	4R	-0.5	0.6	4.4	4.5	
	4L	-3.8	-3.0	-2.8	5.6	
	4L	-7.3	-0.3	-3.1	7.9	
Mean (mm)		-4.4	-2.9	5.1	10.0	
Median (mm)		-3.7	-2.5	5.6	11.0	

Navigation system accuracy. Deviation between CT and EBUS position in the EM field for five mediastinal lymph nodes. AA = aortic arch. PA = pulmonary artery. AV = azygos vein.

### Clinical feasibility

**Fig 6:**
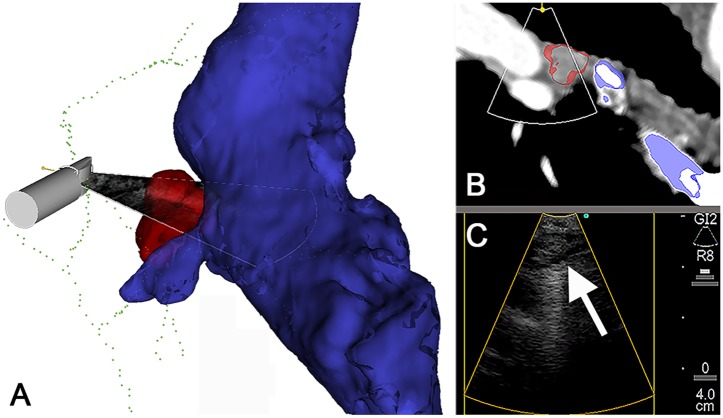
Navigated EBUS-TBNA used to assess operability in patient 3. (A) Preoperative CT indicated a non-curative setting due to a probable lymph node metastasis proximal to the azygos vein (blue). Intraoperative multimodal images correctly placed the lymph node (red) to a different level than CT, concluding with less advanced disease. For improved overview of the vasculature, the airway CT models were not included in this visualization. (C) Real-time EBUS imaging the azygos vein and 10R lymph node (arrow). (B) Corresponding CT plane with US sector position indicated (overlaid the CT).

The maneuverability of the experimental EBUS bronchoscope did not differ from a conventional EBUS-bronchoscope. None of the experimental components interfered with the OR workflow. All the predefined mediastinal lymph nodes were effectively approached guided by EM navigation during the target localization phase (Figs 4 and 6).

Representative specimen was obtained in 13/19 TBNA punctures from six different lymph nodes. The overall yield per targeted lymph node was 6/6 (100%), confirmed on-site by identification of lymphoid and/or atypical cells in the smear (Table 1). The mean procedure time was 24 min 38 sec (16–42 min), including initial video-bronchoscopy, application of topical anesthetics, image-to-patient registration and sampling (Table 1). None of the procedures had to be stopped prematurely due to patient discomfort. No procedure related adverse events or unexpected incidents occurred. All patients were provided with a diagnostic conclusion after the study, and none had to undergo re-endoscopy.

### Technical feasibility

#### Image fusion, multimodal visualizations

The image-to-patient registration algorithm functioned to its purpose. The preoperative CT images could be automatically registered to the patient during airway anesthesia in all the study procedures. EM position tracking was feasible in spontaneously breathing patients, and did not interfere with endoscopy. EBUS images acquired by the tracked probe could be imported into the navigation software during all procedures. Multiple visualization modalities: EBUS, preoperative CT and segmented CT models (airways, targets, vessels) were successfully fused in the navigation software and displayed with the position of the EBUS bronchoscope (Figs 4 and 6) in all patients. There were several user interface and visualization options, each had high quality and could be used for procedure guidance and documentation (Figs 4 and 6).

#### Bronchoscope guiding

The EM sensors on the experimental bronchoscopes were traceable throughout all procedures, allowing continuous control of bronchoscope position in the airways. The multimodal image display gave a clear overview visualization of the target lymph nodes and the surrounding anatomy (Figs 4 and 6). EM navigation facilitated detection of the correct mediastinal lymph node level. EBUS helped visualize and select the predefined lymph node from CT, and provided TBNA guidance. The navigation display enabled a quick and precise reorientation of probe position after each needle puncture, preparing for the next TBNA.

#### Navigation system accuracy

The mean position deviation observed between EBUS images and CT center position for five mediastinal lymph nodes (in total 11 measurements from four patients) was 10.0 (4.5–17.6 mm) (Table 2).

## Discussion

The feasibility of navigated EBUS was successfully demonstrated in humans according to the predefined clinical and technical feasibility parameters. Using EM tracking and CT/EBUS image fusion, the intraoperative position of a prototype convex EBUS-probe could be displayed with a mean navigation accuracy of 10.0 mm (Figs 4 and 6).

Real-time tracking and fusion of EBUS images in humans has not been reported previously, and no commercial providers offer actual EM tracking for any type of EBUS-probe. Sequential EBUS and EM navigation is possible using trading systems and radial array US miniprobes designed for guidance to peripheral lung lesions. The tracking probe in the bronchoscope’s working channel is then replaced with the US miniprobe once at the region of interest [9, 12, 13, 23–30].

A research navigation system presented by Luo et al implied EM tracking and position measurements from external sensors on an US miniprobe [31]. The reported tracking accuracy was 2.6 mm [31], yet to be validated in clinical studies [32].

Advanced image-guiding for convex probe EBUS is only available in research solutions. Zang et al have presented a prototype that integrates images from the EBUS-probe into their own virtual bronchoscopy system [33]. An optimal route to target is calculated based on preoperative CT images. The EBUS images are synchronized with CT data during bronchoscopy [33].

Another image guiding system fusing convex probe EBUS with preoperative images has been subjected to limited testing in humans [34–37]. Procedure guiding is possible if targets are defined and CT models extracted in advance. Virtual bronchoscopy is used for bronchoscope positioning, which but could be less robust than EM tracking in cases of bronchoscope lens clouding [34–36]. In our study, CT models were not mandatory to perform navigated EBUS. Sampling could be customized based on real-time video and US findings.

In favour of our EBUS navigation system it is video-independent, user-friendly and provides simultaneous position control and image guidance. All image and position data are stored, so a complete procedure replay is possible. This is currently not a feature in other EBUS-TBNA systems. Although software and hardware study components are in-house solutions, the study results can be reproduced using open access software (www.custusx.org) and freely available image data (https://datadryad.org/) [15].

The external EM tracking sensor solution was vulnerable, but left a free working channel, allowed continuous position tracking and kept a constant relation between the EM sensor and the US probe [21]. An internal sensor has previously proven technically difficult, and a loose sensor in the working channel would hinder TBNA [38]. A future solution could be a reusable clip-on device that could be removed during cleaning.

Our method for EM navigated EBUS proved feasible in a previous phantom experiment [20, 39, 40]. A mean navigation accuracy of 10.0 mm in humans is not discouraging. Navigation error could have been inflicted at several stages of the image fusion process by CT-to-patient mismatch, patient and respiratory movement, tissue movement caused by the EBUS-probe and by EM devices and metal objects in the bronchoscopy suite (Fig 1) [7, 15, 16, 19]. While imaging a paratracheal lymph node an increasing position deviation of the EBUS images was observed, resulting in a maximum navigation error of 17.6 mm (Table 2). Tissue displacement inflicted by the bronchoscope was the probable cause [7]. The natural movement of mediastinal lymph nodes is variable through the respiratory cycle [41, 42]. Larger studies are required before concluding on the in vivo navigation accuracy. An OR environment with limited EM disturbances, reduced time span from CT to endoscopy, and improved software error correcting schemes can reduce navigation error in the future.

Accuracy calculations were based on a manual segmentation of CT models and alignment from US to CT volumes, possibly introducing experimental error. This could be minimized by applying user independent segmentation algorithms, standardized CT protocols and letting more than two persons perform this part of the study (Figs 4–6) [22]. The EBUS images and position coordinates were acquired ahead of TBNA, so the technical outcomes are valid only for the target localization phase. Traceable sampling tools must be used to assess navigation accuracy during TBNA.

All patient investigations were according to the national lung cancer guidelines, using a simple, reproducible OR set-up in a realistic, clinical setting [43]. The clinical outcomes were within the usual range in our bronchoscopy unit [44] (Table 1). All but one target lymph node (Table 2), were successfully visualized by the navigation system (Figs 4 and 6). Combined EBUS/ENB was useful for effective and precise bronchoscope guidance and image documentation. The CT of patient 3 indicated a non-curative setting (Fig 6). Intraoperative multimodal visualizations correctly placed the target lymph node to a lower stage level, confirmed by EBUS (Fig 6). The patient could be offered radical treatment, indicating a clinical value of the combined modalities.

Our image guiding system has previously proved reliable [15, 16, 39]. Robustness seemed good also in the current study, based on repeated positional measurements (Table 2). The future system usability depends on optimized error correction, especially respiratory motion compensation. Regardless of patient movement, progress of disease and navigation error, EBUS shows the true anatomy. Lymph node coordinates in the CT/navigation system should therefore ideally be compared to the US images.

Improving success rates for EBUS-TBNA might be challenging [1]. However, the number and difficulty of procedures will escalate following routine mediastinal mapping. Previous reports on procedure performance might not apply. High-end ultrasound processors and reduced diameter of the EBUS bronchoscope can increase the diagnostic reach of convex probe EBUS. Additional image modalities during EBUS-TBNA can facilitate precise, accurate, efficient and minimally invasive procedures. Intraoperative CT-based EM guidance can aid the initial 3D orientation of a lesion’s level, location, shape and size. EMN can support the localization and distinction of lesions when US landmarks are ambiguous. The combined EMN/EBUS system can be used for procedure planning, documentation and training. EMN also seems to make the localization of each target level quicker, of particular interest in procedures with multiple targets.

Our navigation system makes new visualization techniques possible. EMN allows both 3D EBUS and incorporation of modern image modalities such as PET-CT and PET-MRI [39, 45]. Future areas of priority will be to improve tool tracking and navigation visualizations, and to reduce error. A more detailed human pilot study is ongoing (NCT 02745002).

There are a few study limitations. A complete mediastinal mapping was considered futile in patients 1–3, probably reducing procedure duration. The specific contribution of multimodal image guiding to the clinical outcomes could not be concluded. Still, the supplement of EMN could be of importance for the yield and efficiency of EBUS-TBNA [11]. The navigation system was tested in a low number of patients with centrally positioned lymph nodes, in one single bronchoscopy unit, and with just one extra image modality (CT) fused into the navigation system. Study results should therefore be validated in patients with non-malignant conditions, involving several operators and OR environments.

## Conclusions

The feasibility of EM navigated EBUS-TBNA was successfully demonstrated in four lung cancer patients with respect to predefined outcomes involving technical functionality, accuracy, procedure time, safety and sample adequacy. The new navigation system offers a fast and precise guiding to the level of target, procedure overview and documentation. Navigated EBUS has a great potential for further image quality refinement, accuracy improvement and fusion of additional image modalities. Larger studies are required to assess the effect of addition of EM navigation on EBUS-TBNA efficacy, and extended human pilot studies are underway.
